# Automatic Lesion Morphometrics Reliably Predict Sensorimotor Recovery After Spinal Cord Injury: A Longitudinal, Multicenter Neuroimaging Study

**DOI:** 10.1002/acn3.70569

**Published:** 2026-11-05

**Authors:** Dario Pfyffer, Jan Valošek, Lynn Farner, Enamundram Naga Karthik, Gergely David, Norbert Weidner, Rainer Abel, Doris Maier, Armin Curt, Margret Hund‐Georgiadis, Mirko Aach, Michael Baumberger, Julien Cohen‐Adad, Patrick Freund, the Nogo Inhibition in Spinal Cord Injury Study Group, Rainer Abel, Kerstin Rehahn, Mirko Aach, Klaus Röhl, Norbert Weidner, Marion Saur, Nikolaus Weiskopf, Doris Maier, Andreas Badke, Margret Hund‐Georgiadis, Michael Baumberger, Armin Curt, Jesus Benito‐Penalva, Jiri Kriz

**Affiliations:** ^1^ Spinal Cord Injury Center Balgrist University Hospital, University of Zurich Zurich Switzerland; ^2^ Department of Anesthesiology, Perioperative and Pain Medicine Stanford University School of Medicine Palo Alto California USA; ^3^ NeuroPoly Lab, Institute of Biomedical Engineering Polytechnique Montreal Montreal Quebec Canada; ^4^ Department of Neurosurgery and Spine Surgery and Department of Neurology, Faculty of Medicine and Dentistry Palacký University Olomouc Olomouc Czechia; ^5^ Department of Biomedical Engineering University Hospital Olomouc Olomouc Czechia; ^6^ Mila ‐ Quebec AI Institute Montreal Quebec Canada; ^7^ Spinal Cord Injury Center Heidelberg University Hospital Heidelberg Germany; ^8^ Medical Faculty Heidelberg Heidelberg University Heidelberg Germany; ^9^ Clinic for Paraplegia Klinikum Bayreuth Bayreuth Germany; ^10^ Spinal Cord Injury Center BG Unfallklinik Murnau Murnau Germany; ^11^ Clinic of Neurorehabilitation and Paraplegiology REHAB Basel Basel Switzerland; ^12^ Department of Spinal Cord Injuries BG University Hospital Bergmannsheil Bochum Germany; ^13^ Swiss Paraplegic Centre Nottwil Switzerland; ^14^ Functional Neuroimaging Unit, CRIUGM Université de Montréal Montreal Quebec Canada; ^15^ Centre de Recherche du CHU Sainte‐Justine Université de Montréal Montréal Quebec Canada; ^16^ Max Planck Institute for Human Cognitive and Brain Sciences Leipzig Germany; ^17^ Wellcome Trust Centre for Neuroimaging, Queen Square Institute of Neurology University College London London UK

**Keywords:** lesion morphometry, outcome prediction, SCIsegV2, sensorimotor recovery, spinal cord injury, structural MRI

## Abstract

**Objective:**

To evaluate automatic magnetic resonance imaging (MRI)‐derived lesion morphometrics against manual measurements and their prognostic value for characterizing distinct acute to chronic neurological recovery trajectories after spinal cord injury (SCI).

**Methods:**

We studied a retrospective, multicenter cohort of cervical and thoracolumbar SCI patients who underwent routine clinical MRI at 3–4 weeks post‐SCI and neurological examinations over 6 months. Lesion severity was quantified using both established manual measurements and a novel automatic approach. We assessed the between‐method agreement of MRI‐derived lesion morphometrics, including lesion length, lesion width, and tissue bridge width, and their ability to stratify patients according to 6‐month motor and sensory outcomes. Data‐driven prediction models were used to identify clinically meaningful lesion measures and their respective thresholds, differentiating subgroups of specific recovery profiles.

**Results:**

Eighty‐three patients from eight sites were included. Automatically derived lesion morphometrics showed moderate to strong agreement with established manual measures (Spearman's ρ: 0.540–0.821, *p* < 0.001), capturing inter‐individual differences in lesion severity and demonstrating comparable prognostic value. Lesion width represented the strongest predictor of 6‐month neurological outcomes, splitting patients into recovery‐specific subgroups. Patients with a smaller lesion width (threshold: 5.88–6.41 mm) demonstrated higher 6‐month sensorimotor scores and superior recovery trajectories, while tissue bridges (threshold: 0.17 mm) further refined motor prediction‐based stratification of more severely affected patients.

**Interpretation:**

Automatic MRI measures of lesion severity hold promise as scalable, time‐efficient in vivo biomarkers of SCI prognosis, with potential for clinical adoption to guide treatment decisions and multicenter trial planning.

**Abbreviations**
AIS: ASIA Impairment Scale
ASIA: American Spinal Injury Association
ISNCSCI: International Standards for the Neurological Classification of Spinal Cord Injury
LEMS: lower extremity motor score
MRI: magnetic resonance imaging
NISCI: Nogo Inhibition in Spinal Cord Injury
PVE: partial volume effect
SCI: spinal cord injury
SCT: Spinal Cord Toolbox
T2w: T2‐weighted
UEMS: upper extremity motor score
URP‐CTREE: unbiased recursive partitioning conditional inference tree

## 1 Introduction

The complex neuropathological processes underlying severe neurological impairments following spinal cord injury (SCI) vary widely across subjects and largely depend on the severity of the injury and subsequent secondary changes [1]. Patients who present with a similar clinical status at hospital admission, as assessed by the International Standards for the Neurological Classification of SCI (ISNCSCI) [2], may exhibit different trajectories of sensorimotor recovery [3]. This can be attributed to the ISNCSCI's limitations, including assessment variability across clinicians, inability to capture subtle changes, and limited information about the underlying structural damage that constrains recovery. This significantly complicates rehabilitation planning and clinical outcome prediction.

Magnetic resonance imaging (MRI), routinely acquired in the sub‐acute phase after SCI, offers non‐invasive visualization of intramedullary pathology and can be utilized to obtain prognostic biomarkers in SCI. Among the most promising are T2‐weighted (T2w) MRI‐derived quantitative measures of the lesion extent and amount of spared neural tissue. These include midsagittal rostrocaudal lesion length, transverse lesion width, and preserved spinal tissue bridges [4, 5]. These lesion morphometrics have been shown to correlate with motor and sensory recovery in both traumatic and non‐traumatic SCI, beyond baseline clinical measures alone [6, 7, 8, 9, 10, 11, 12]. Tissue bridges, in particular, have been consistently associated with neurological improvement across independent cohorts and were incorporated in a recent multicenter clinical trial [13].

MRI‐derived measures are usually assessed manually on a single midsagittal slice, which, while clinically informative, is time‐intensive, subject to intra‐ and inter‐rater variability, and difficult to scale across centers. In addition, clinically acquired MRI scans in SCI typically have relatively thick slices (in order of millimeters), which complicates the midsagittal slice selection and limits reproducibility. Recent advances in computer vision and artificial intelligence have enabled the automatic segmentation of SCI lesions from conventional MRI scans, including our deep learning tool SCIseg [14, 15]. This can be leveraged for various downstream analyses in clinical and research settings, such as standardized quantification of MRI‐derived lesion measures [15, 16, 17]. However, there is a lack of automatic methods for calculating lesion morphometrics on the midsagittal slice, preventing a direct comparison and validation of an automatic approach against the gold standard, a manual lesion assessment. Further, whether automatically derived measures preserve the ability to predict sensorimotor recovery post‐SCI—an essential feature for clinical adoption—has yet to be demonstrated.

In this multicenter longitudinal study, we compared automatic MRI‐derived lesion morphometrics (i.e., midsagittal lesion length, lesion width, and tissue bridges) with manually measured ones in a heterogeneous cohort of acute cervical and thoracolumbar SCI patients. Since agreement with manual measurements does not directly imply equivalent prognostic value of automatic metrics, we compared both approaches in predicting sensorimotor outcomes at 6‐month post‐SCI using data‐driven prediction‐based stratification models. To further test the clinical relevance, we determined whether the identified cut‐offs of automatic lesion measures can define distinct subgroups of specific neurological recovery profiles over 6 months follow‐up. Our developed method is open‐source, integrated into the Spinal Cord Toolbox (SCT) [18], and can be seamlessly used for automatic computation of lesion measures to characterize SCI severity from clinical MRI scans.

## 2 Materials and Methods

### 2.1 Study Design and Subjects

This longitudinal, multicenter (*n* = 8 sites), retrospective study included 83 patients with SCI from two cohorts. Forty‐six were admitted to the Balgrist University Hospital, Zurich, Switzerland (May 2002–February 2023), and 37 were in the placebo arm of the Nogo Inhibition in Spinal Cord Injury (NISCI) interventional trial assessing the efficacy and safety of NG101 (ClinicalTrials.gov ID: NCT03935321).

The research was conducted in accordance with the Declaration of Helsinki. All study participants gave written, informed consent at hospital admission for their data to be used for research purposes. Inclusion criteria were cervical or thoracolumbar SCI (vertebrae C1–L2), MRI assessment at imaging baseline (Zurich: 45.1 ± 27.0 days [range: 12.0–133.0], NISCI: 19.8 ± 4.8 days [11.0–33.0]), and serial clinical examinations at clinical baseline (Zurich: 8.2 ± 5.0 days [1.0–24.0], NISCI: 18.1 ± 6.3 days [2.0–28.0]), 1‐month (Zurich: 29.8 ± 6.0 days [17.0–41.0], NISCI: 36.0 ± 4.2 days [25.0–42.0]), 3‐month (Zurich: 84.1 ± 9.8 days [52.0–99.0], NISCI: 106.1 ± 6.3 days [92.0–118.0]), and 6‐month (Zurich: 173.6 ± 11.6 days [138.0–213.0], NISCI: 191.2 ± 16.5 days [165.0–257.0]) follow‐up. Exclusion criteria were concurrent traumatic brain injury, preexisting neurological or psychiatric history, MRI contraindications, or MRI assessment within the first 10 days post‐SCI. Detailed inclusion and exclusion criteria for both cohorts can be found in our previous studies [6, 12, 13, 14, 17].

### 2.2 Clinical Assessment

Patients underwent neurological examinations performed by physicians or physical/occupational therapists using the ISNCSCI protocol [2]. The ISNCSCI protocol comprises bilateral sensorimotor testing of 20 key muscle groups graded 0–5 (total motor score 0–100 points, consisting of an upper extremity motor score [UEMS; 0–50 points] and lower extremity motor score [LEMS; 0–50 points]) and 28 dermatomes graded 0–2 on each side of the body (pinprick and light touch tests, 112 points each). According to the degree of neurological function, patients were classified into AIS grades A (complete sensorimotor impairment), B (complete motor impairment and incomplete sensory impairment), C (incomplete motor impairment with lower muscle ratings), D (incomplete motor impairment with higher muscle ratings), or E (no functional impairment). The UEMS was not analyzed since patients with a thoracolumbar SCI did not show any deficits in UEMS (i.e., maximum score).

### 2.3 Image Acquisition

Patients were scanned at 1.5 T (*n* = 33, 39.8%) or 3.0 T (*n* = 50, 60.2%) on a Siemens (Siemens Healthcare, Erlangen, Germany), Philips (Philips Healthcare, Best, Netherlands), or Toshiba (Canon Medical Systems Cooperation, Otawara, Tochigi, Japan) system in a supine head‐first position using a 16‐channel or a 32‐channel receive spine coil integrated in the table. The conventional clinical MRI protocol consisted of anatomical sagittal T1‐weighted and sagittal and axial T2w sequences of the cervical/thoracolumbar spinal cord, and was centered on the lesion level. For the purpose of this study, sagittal T2w scans were used. For the Zurich dataset, the sagittal T2w in‐plane resolution ranged from 0.43–0.89 mm (0.59 ± 0.10 mm), and the slice thickness ranged from 2.20–4.83 mm (3.15 ± 0.65 mm). For the NISCI dataset, the sagittal T2w in‐plane resolution ranged from 0.25–0.81 mm (0.53 ± 0.14 mm), and the slice thickness ranged from 2.70–3.30 mm (2.84 ± 0.21 mm). MRI scans were converted from Digital Imaging and Communications in Medicine (DICOM) format to Neuroimaging Informatics Technology Initiative (NIfTI) format and organized according to the Brain Imaging Data Structure (BIDS) standard [19]. During this curation process, all sensitive patient information was removed.

### 2.4 Manual Lesion Quantification

Figure 1A illustrates the manual measurements of the lesion morphometry. The midsagittal slice was identified by first counting all slices where the spinal cord was visible at the level of the lesion. The middle slice was then selected; if the number of slices was even, the slice with the larger spinal cord width of the two middle slices was used. Lesions, appearing as T2w signal abnormalities (hyper−/hypointense voxels corresponding to primary contusions, cystic cavities, secondary cytotoxic edema, or hemorrhage), were manually annotated by two raters (D.P. and L.F.), who were blinded to the automatic measurements, using JIM software (version 7.0, Xinapse Systems, Aldwincle, UK). We note that hyperintense edema typically resolves within the first 2–3 weeks post‐SCI, after which the cystic cavity becomes demarcated, enabling reliable quantification of the lesion extent and tissue bridges spanning the spared neural tissue, even in proximity of metal artifacts [5, 6, 9].

**FIGURE 1:**
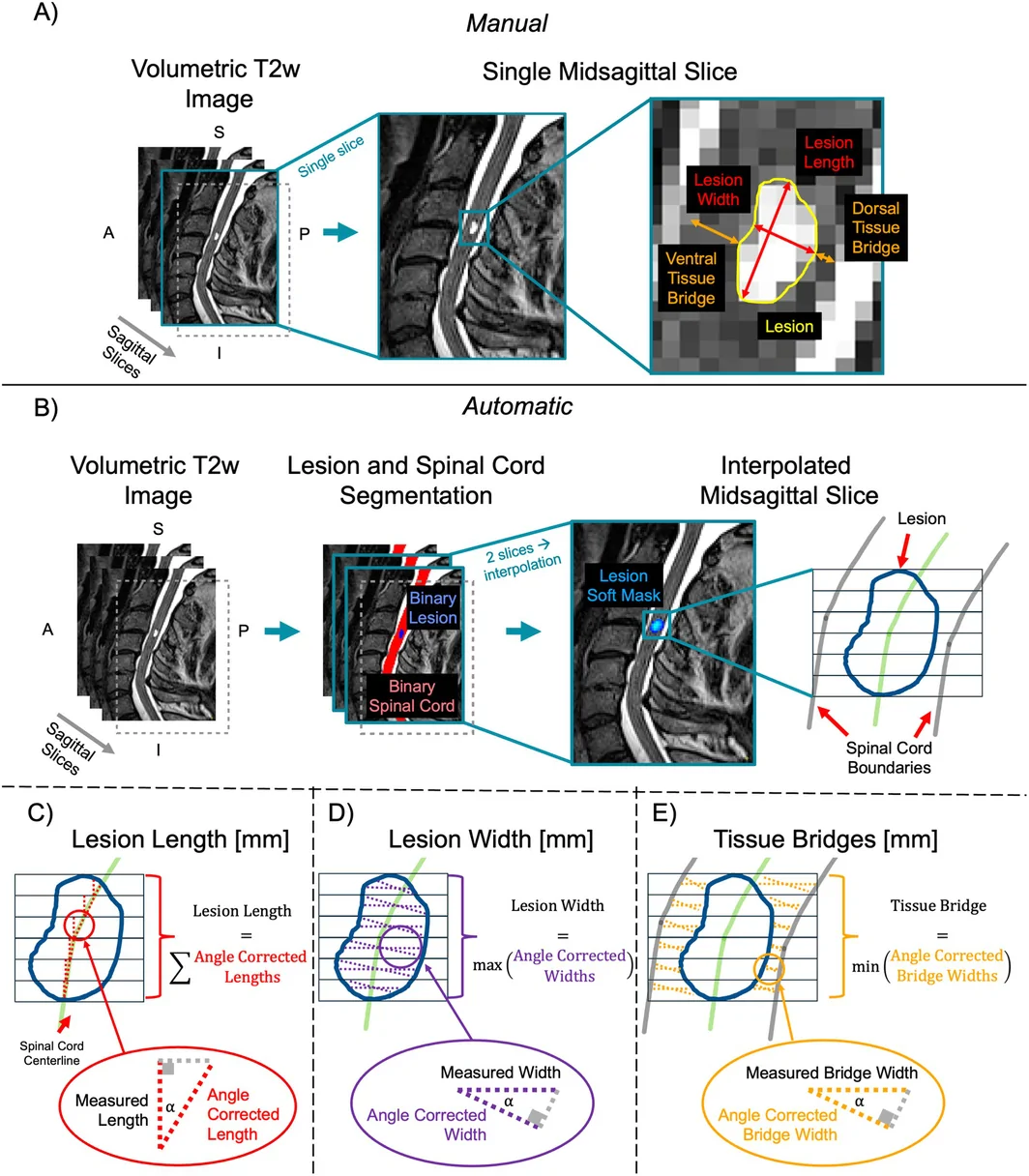
Magnetic resonance imaging (MRI)‐derived measurements of the lesion morphometry. T2‐weighted (T2w) image of a patient with spinal cord injury (SCI) with an intramedullary lesion appearing as a hyperintense signal at the vertebral level of C3/C4. (A) *Manual measurements*: A single mid‐sagittal slice is used to manually annotate and measure lesion length, lesion width, and ventral and dorsal tissue bridges, summing up to the total tissue bridge width. (B) *Automatic measurements*: A volumetric (i.e., 3D) T2w image is used to automatically segment spinal cord (red) and lesion (blue) binary masks. These segmentation masks are used to obtain the interpolated midsagittal slice with a soft (i.e., non‐binary) lesion mask (only the mask contour is shown for clarity) based on the sagittal slices closest to the lesion center of mass; see Data S2 for details. Schematic of automatic (C) lesion length, (D) lesion width, and (E) ventral and dorsal tissue bridges calculation. Length is measured along the superior–inferior (SI) axis of the spinal cord as the sum of slice thicknesses, lesion width is defined as the maximum width across axial slices, and ventral and dorsal tissue bridges are defined as the minimum bridge width across axial slices. The angle correction is used to account for the curvature of the spinal cord.

Lesion length and width were measured manually, parallel to the spinal cord in the superior–inferior (SI) axis and perpendicular to the spinal cord in the anterior–posterior (AP) axis, respectively, accounting for the angle between the spinal cord and the axial plane [7, 9]. Ventral and dorsal tissue bridges were defined as the minimal distances between the T2w‐hyperintense lesion and the hyperintense cerebrospinal fluid of the spinal canal. The total tissue bridge width is defined as the sum of ventral and dorsal tissue bridges.

### 2.5 Automatic Lesion Quantification

To automatically measure the lesion morphometry, we developed a method that automatically obtains a midsagittal slice by interpolating two sagittal slices closest to the center of the spinal cord at the level of injury. This resulted in a soft (i.e., non‐binary) lesion mask to account for partial volume effects (PVE) and increase the precision of lesion morphometry measurements (Figure 1B and Data S2). Rostrocaudal midsagittal lesion length was measured along the SI axis as the sum of slice thicknesses weighted by the lesion softness (Figure 1C). Lesion width was measured along the AP axis for each axial slice as the sum of non‐zero voxel values (i.e., voxels occupied by the lesion), and the final midsagittal width was defined based on the axial slice with the maximum lesion width (Figure 1D). Ventral and dorsal midsagittal tissue bridges were quantified as the weighted width of spared tissue along the AP axis at the minimum distance from the intramedullary lesion edge to the boundary between the spinal cord and cerebrospinal fluid (Figure 1E). Ventral and dorsal midsagittal tissue bridges were then summed to the total width of tissue bridges. All measures were corrected by the angle between the spinal cord centerline and the axial plane to account for the curvature of the spinal cord. In patients with multiple lesions, the lengths were summed for a composite length, the largest width across all lesions was taken as the maximal lesion width, and the minimum ventral and dorsal tissue bridges across all lesions were used.

The proposed method was used to measure lesion morphometry from lesion and spinal cord masks automatically obtained using the deep learning‐based SCIsegV2 algorithm [14, 15] as part of SCT [18] v7.0.

### 2.6 Statistical Analysis

Statistical analysis was performed using the SciPy Python library [20] (version 1.14.1). Manual and automatic lesion measurements (i.e., midsagittal lesion length, lesion width, and total tissue bridges) were compared using correlation and agreement analyses. The Shapiro–Wilk test was used to test the null hypothesis that the data was drawn from a normal distribution. Associations between manual and automatic estimates were assessed using Spearman's rank correlation coefficient. Agreement between methods was evaluated using Bland–Altman analysis. All analyses were performed across the full cohort using paired manual and automatic measurements for each metric. A *p*‐value < 0.05 indicates statistical significance.

Further, we applied unbiased recursive partitioning conditional inference tree (URP‐CTREE) models, implemented in the *party* package in R (version 3.4.3), as described in detail previously [21] and applied extensively in SCI [6, 12, 22, 23, 24, 25, 26]. These prediction‐based stratification models identify predictors and corresponding cut‐offs, partitioning a neurologically heterogeneous patient cohort into more homogeneous subgroups with maximized differences in predefined clinical endpoints. In short, we used a combination of baseline imaging measures (lesion length, lesion width, tissue bridge width), demographic covariates (age, sex, center), and the neurological level of injury (NLI) as predictors, and 6‐month clinical scores as endpoints in our URP‐CTREE models. Clinical outcome measures comprised ISNCSCI‐derived LEMS, total motor score, pinprick score, and light touch score. To further compare manual and automatic lesion quantification and validate the URP‐CTREE findings, each algorithm was run twice, with imaging predictors including (i) manually derived lesion measures and (ii) automatically derived lesion measures. URP‐CTREEs were also modeled on a sub‐cohort of patients with MRI assessed beyond 3 weeks post‐SCI to investigate the robustness of the models against timing of MRI as a potential confound.

Longitudinal changes in clinical scores were analyzed using mixed‐effects models. For each outcome, a single model was fitted with fixed effects of time (days post‐injury), group (defined by baseline lesion‐metric cut‐offs identified by URP‐CTREE models), and their interaction (score~days × group) to estimate group‐specific intercepts and recovery slopes. To accommodate nonlinear recovery [27], logarithmic time since injury was used [28]. Random effects included participant‐specific intercepts and slopes with respect to time, accounting for repeated measures and inter‐individual variability in recovery trajectories. Model‐derived fixed effects were used to visualize fitted trajectories alongside individual longitudinal profiles.

Of 83 patients, 76 had their first neurological examination at baseline, while the first assessment of the other subjects was performed at 1‐month (*n* = 6) or 3‐month (*n* = 1) follow‐up. Eight patients had missing data at 6‐month post‐SCI (i.e., *n* = 75 for URP‐CTREE). AIS grades were not available for some patients (baseline: *n* = 8, 1‐month: *n* = 4, 3‐month: *n* = 4, 6‐month: *n* = 8) due to the lack of sacral examination.

## 3 Results

### 3.1 Clinical and Imaging Characteristics

The cohort consisted of 83 patients with cervical (*n* = 63) or thoracolumbar (*n* = 20) SCI (17 females, 66 males; age [mean ± SD] 49.5 ± 17.6 years). Distribution of sex, age, AIS grade over time, and NLI at baseline can be found in Figure 2. Specifically, patients' AIS grades improved from baseline (AIS A: 18, AIS B: 10, AIS C: 22, AIS D: 25, unknown: 8) to 1‐month (AIS A: 15, AIS B: 11, AIS C: 12, AIS D: 41, unknown: 4), 3‐month (AIS A: 9, AIS B: 9, AIS C: 13, AIS D: 48, unknown: 4), and 6‐month (AIS A: 11, AIS B: 7, AIS C: 8, AIS D: 47, AIS E: 2, unknown: 8). Clinical scores increased over 6 months follow‐up, with plateauing changes from 3‐ to 6‐month post‐SCI (Table 1).

**FIGURE 2:**
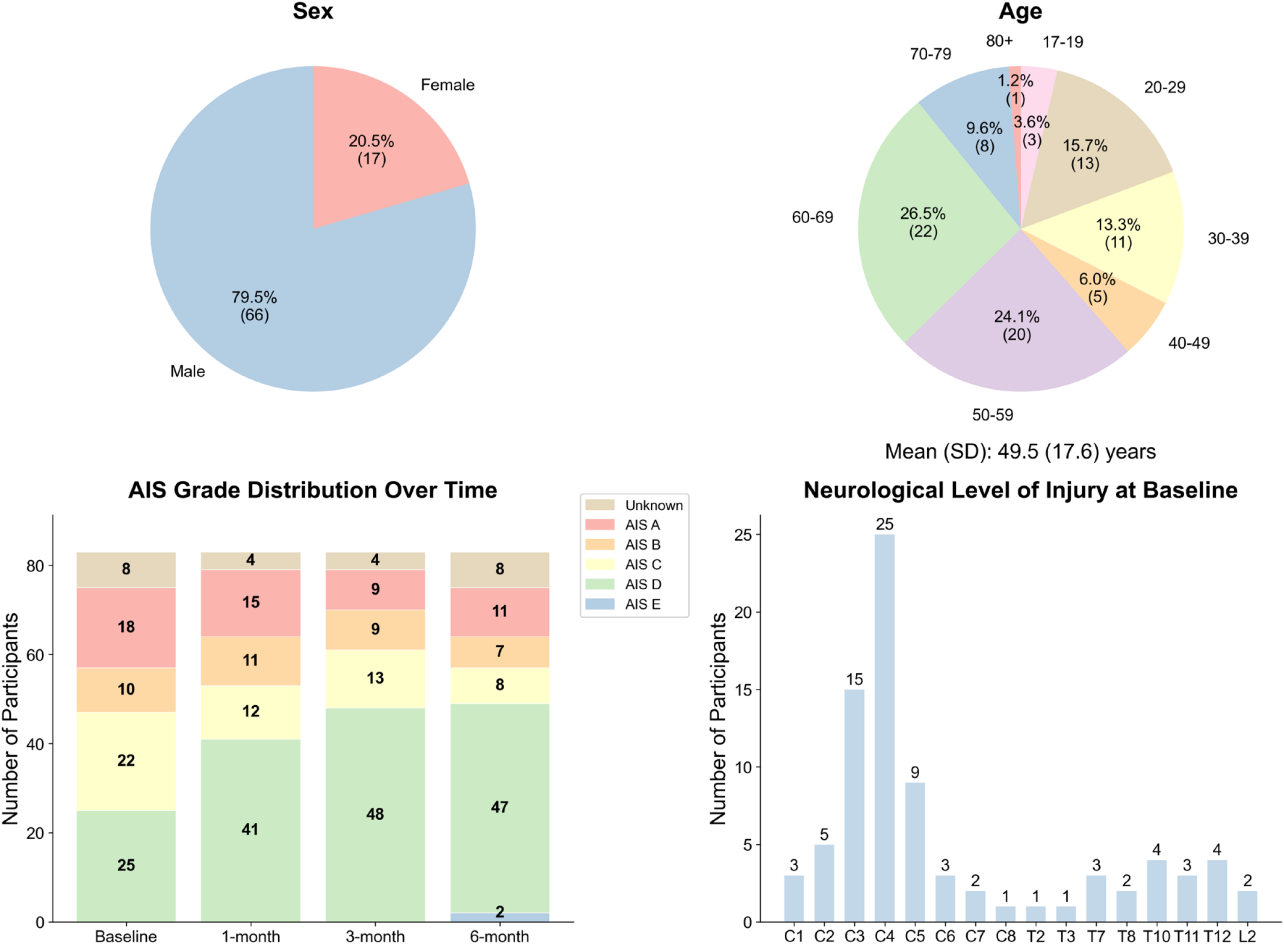
Spinal cord injury (SCI) patient demographics and injury characteristics. Top panel: Proportions of sex and age across the cohort. Bottom panel: Distribution of injury severity at baseline, 1‐month, 3‐month, and 6‐month post‐SCI, and distribution of injury level at baseline. The category “Unknown” for the American Spinal Injury Association Impairment Scale (AIS) reflects patients with unavailable AIS due to a lack of sacral examination.

**TABLE 1:** Clinical scores over the first 6 months following spinal cord injury (SCI).

Clinical score (mean ± SD)	Baseline	1‐month	3‐month	6‐month
LEMS	17.0 ± 17.0	22.4 ± 19.0	27.9 ± 20.3	29.7 ± 21.0
Total motor score	40.8 ± 25.4	52.3 ± 27.6	62.5 ± 29.3	66.2 ± 30.3
Pinprick score	45.4 ± 29.7	54.3 ± 31.6	59.5 ± 32.2	57.8 ± 32.0
Light touch score	64.4 ± 29.1	71.2 ± 27.1	74.2 ± 25.5	73.6 ± 26.9

*Note:* Neurological motor and sensory scores were assessed at baseline, 1‐month, 3‐month, and 6‐month after SCI.

Abbreviation: LEMS, lower extremity motor score.

Automatically obtained midsagittal lesion length, width, and tissue bridges measured 13.5 ± 11.3 mm, 4.6 ± 2.0 mm, and 1.2 ± 1.0 mm, respectively. Manually obtained midsagittal lesion length, width, and tissue bridges measured 19.7 ± 24.9 mm, 5.0 ± 2.3 mm, and 2.0 ± 1.6 mm, respectively.

### 3.2 Comparison Between Manual and Automatic Lesion Morphometrics

Automatic measurements showed moderate to strong agreement with manual measurements for all midsagittal lesion metrics. Lesion length demonstrated a strong positive correlation between methods (ρ = 0.821, *p* < 0.001), while lesion width (ρ = 0.712, *p* < 0.001) and total tissue bridges (ρ = 0.540, *p* < 0.001) showed moderate correlations (Figure 3A). Bland–Altman analyses indicated a small systematic positive bias of manual relative to automatic measurements, with mean differences (manual − automatic) of 6.19 mm for lesion length, 0.41 mm for lesion width, and 0.82 mm for total tissue bridges (Figure 3B).

**FIGURE 3:**
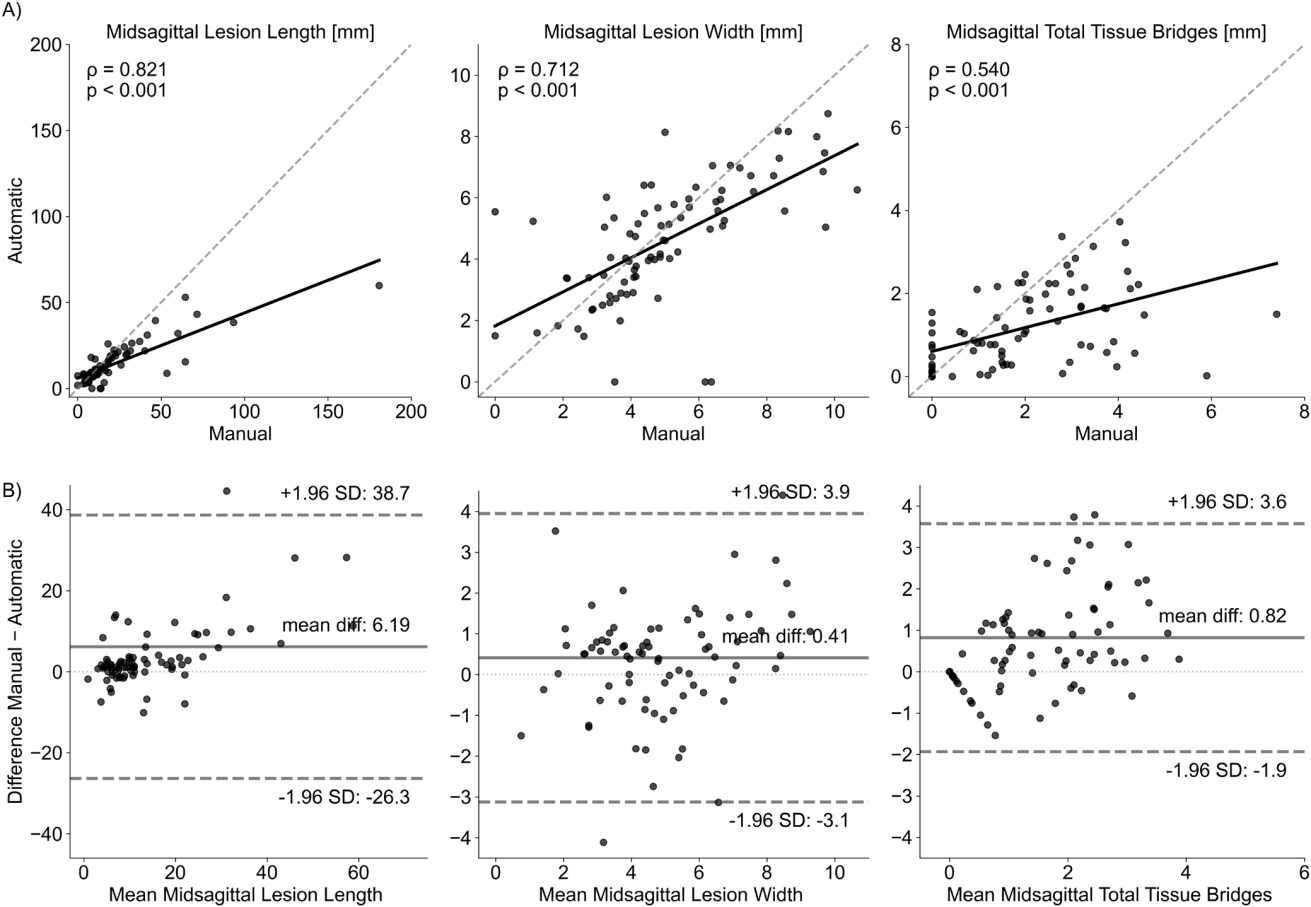
Comparison of manual and automatic measurements of midsagittal lesion characteristics. (A) Scatter plots show the relationship between manual and automatic measurements of midsagittal lesion length, lesion width, and total tissue bridges. Solid black lines represent linear regression fits, and dashed gray lines indicate the line of identity. Spearman correlation coefficients (ρ) and corresponding *p*‐values are reported for each comparison. (B) Bland–Altman plots illustrate agreement between manual and automatic measurements. Solid horizontal lines denote the mean difference (bias), dashed lines indicate the limits of agreement (±1.96 SD), and the dotted line marks zero difference.

### 3.3 Outcome Prediction‐Based Stratification of SCI Patients

From a set of baseline MRI measures (lesion length, lesion width, tissue bridge width; both manually and automatically derived) and demographic variables (age, sex, center), URP‐CTREE models identified significant predictors and respective cut‐offs, separating the SCI patient cohort into subgroups of specific motor and sensory recovery. The algorithm identified manually derived lesion width with a cut‐off of 5.46 mm to split patients into subgroups of higher (≤ 5.46 mm) and lower (> 5.46 mm) 6‐month LEMS and total motor score (Figure 4A, left panel). For automatically derived MRI measures, a similar cut‐off value in lesion width was found to stratify patients into those with higher (≤ 5.88 mm) versus lower (> 5.88 mm) motor outcome (Figure 4A, right panel). Patients with lower 6‐month motor scores (node 3) were further split into subgroups of lower or higher outcomes, according to tissue bridge width being ≤ 0.17 mm or > 0.17 mm, respectively. No demographic variables were determined as predictors.

**FIGURE 4:**
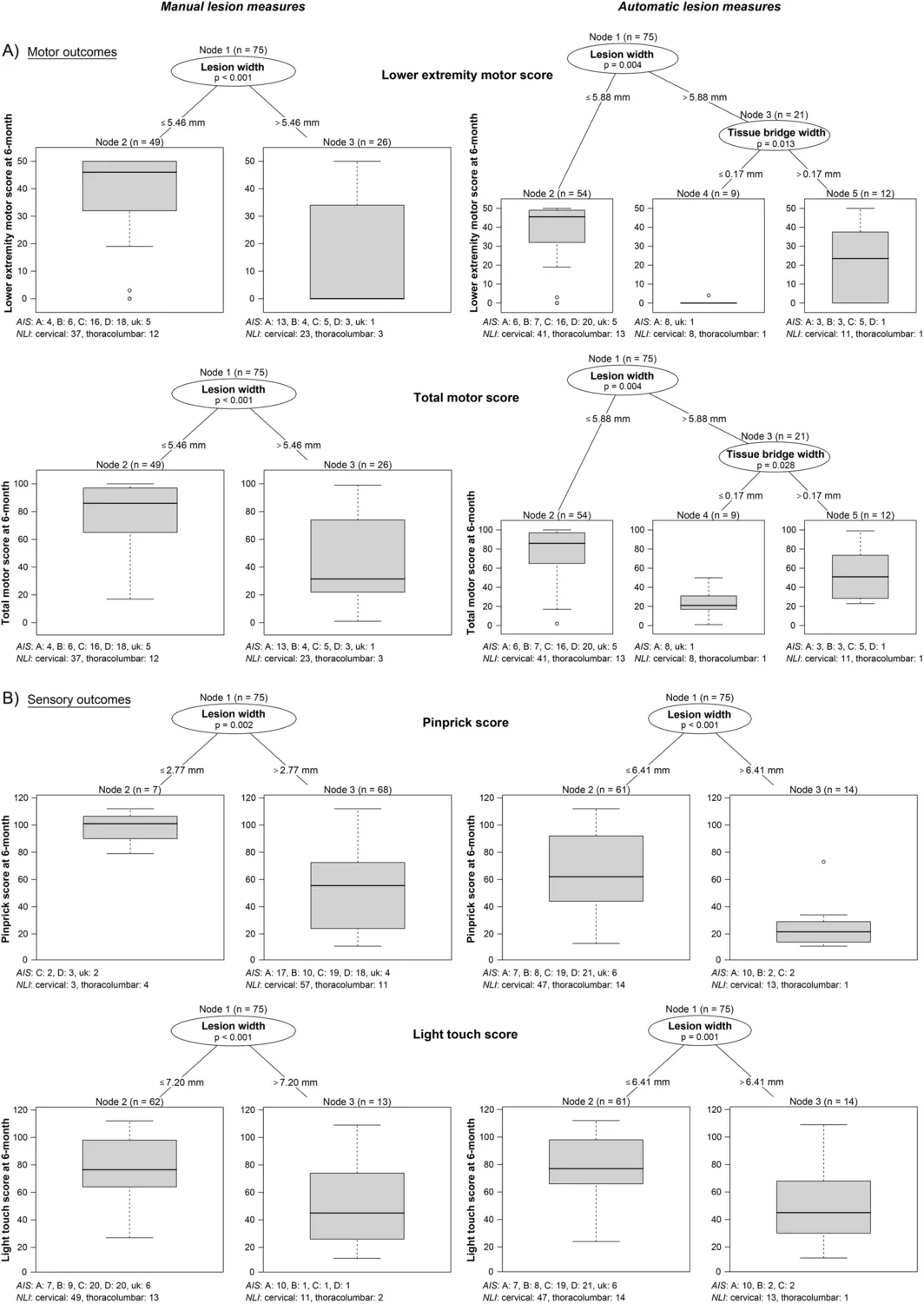
Prediction‐based stratification of patients using baseline lesion measures obtained manually and automatically. Unbiased recursive partitioning conditional inference tree models for 6‐month (A) motor (i.e., lower extremity and total motor score) and (B) sensory (i.e., pinprick and light touch score) outcomes. Left panel: Trees including manual lesion measures as predictors of sensorimotor outcomes. Right panel: Trees including automatic lesion measures as predictors of sensorimotor outcomes. Consistent measures with similar cut‐offs were identified for both methods, validating the automatic method as a reliable tool for quantifying lesion morphometrics and splitting patients into more homogeneous recovery‐specific subgroups. Distribution of 6‐month sensorimotor outcomes is represented by box plots with the median and interquartile range (1st—3rd quartile), and two‐sided error bars. AIS, American Spinal Injury Association Impairment Scale; NLI, neurological level of injury; uk, unknown.

With regard to 6‐month pinprick and light touch score, manually derived lesion width was identified as a predictor splitting patients into a subgroup of higher and lower pinprick (≤ 2.77 mm and > 2.77 mm) and light touch (≤ 7.20 mm and > 7.20 mm) outcome (Figure 4B, left panel). For automatic lesion measures, the algorithm identified lesion width with a cut‐off of 6.41 mm to separate patients into subgroups of higher (≤ 6.41 mm) and lower (> 6.41 mm) scores for both pinprick and light touch (Figure 4B, right panel). No other MRI measures or demographic variables were found to add stratification value.

URP‐CTREE analysis of a sub‐cohort with > 3‐week MRI timepoint (*n* = 47) demonstrated similar thresholds in automatic lesion width as found for the full cohort. Identified cut‐offs measured 5.88 mm for LEMS and total motor score and 5.96 mm for pinprick and light touch scores.

Adding the NLI to the set of predictors, together with MRI measures and demographic variables, did not change URP‐CTREE‐derived predictors and cut‐offs for 6‐month motor outcomes of the full cohort (Data S3). In terms of pinprick score, the algorithm identified the NLI in addition to lesion width, separating patients into subgroups of higher (manual: T1‐L2, automatic: T4‐L2) and lower (manual: C1‐C8, automatic: C1‐T3) outcomes. With regard to 6‐month light touch score, patients with smaller lesion widths were further split according to the NLI into subgroups of higher (T1‐L2) and lower (C1‐C8) outcomes for both the manual and automatic approach. A new manually derived lesion width threshold (≤ 6.50 mm and > 6.50 mm) was identified for 6‐month pinprick score, agreeing with the automatic one. All other thresholds for sensory outcomes were confirmed.

### 3.4 Distinct Subgroups of Longitudinal Motor and Sensory Recovery

Patients with smaller lesion width, based on URP‐CTREE‐derived cut‐offs of automatic measures, showed higher baseline motor and sensory scores and greater improvements over time compared to those with larger lesions (Figure 5). For motor outcomes, patients with a lesion width of ≤ 5.88 mm (green trajectory) demonstrated continuous improvements in both LEMS and total motor score from baseline to 6‐month follow‐up. Among patients with wider lesions (lesion width > 5.88 mm), those with preserved tissue bridges (> 0.17 mm, yellow trajectory) exhibited lower baseline scores but consistent motor recovery, comparable to patients with a lesion width ≤ 5.88 mm. On the other hand, those with minimal or absent tissue bridges (≤ 0.17 mm, red trajectory) showed little to no improvement (Figure 5A). Sensory outcomes followed a similar pattern, with patients with a lesion width ≤ 6.41 mm (green trajectory) achieving higher pinprick and light touch scores at all timepoints. These subjects maintained longitudinal, plateauing gains, while those with wider lesions (red trajectory) demonstrated limited recovery (Figure 5B).

**FIGURE 5:**
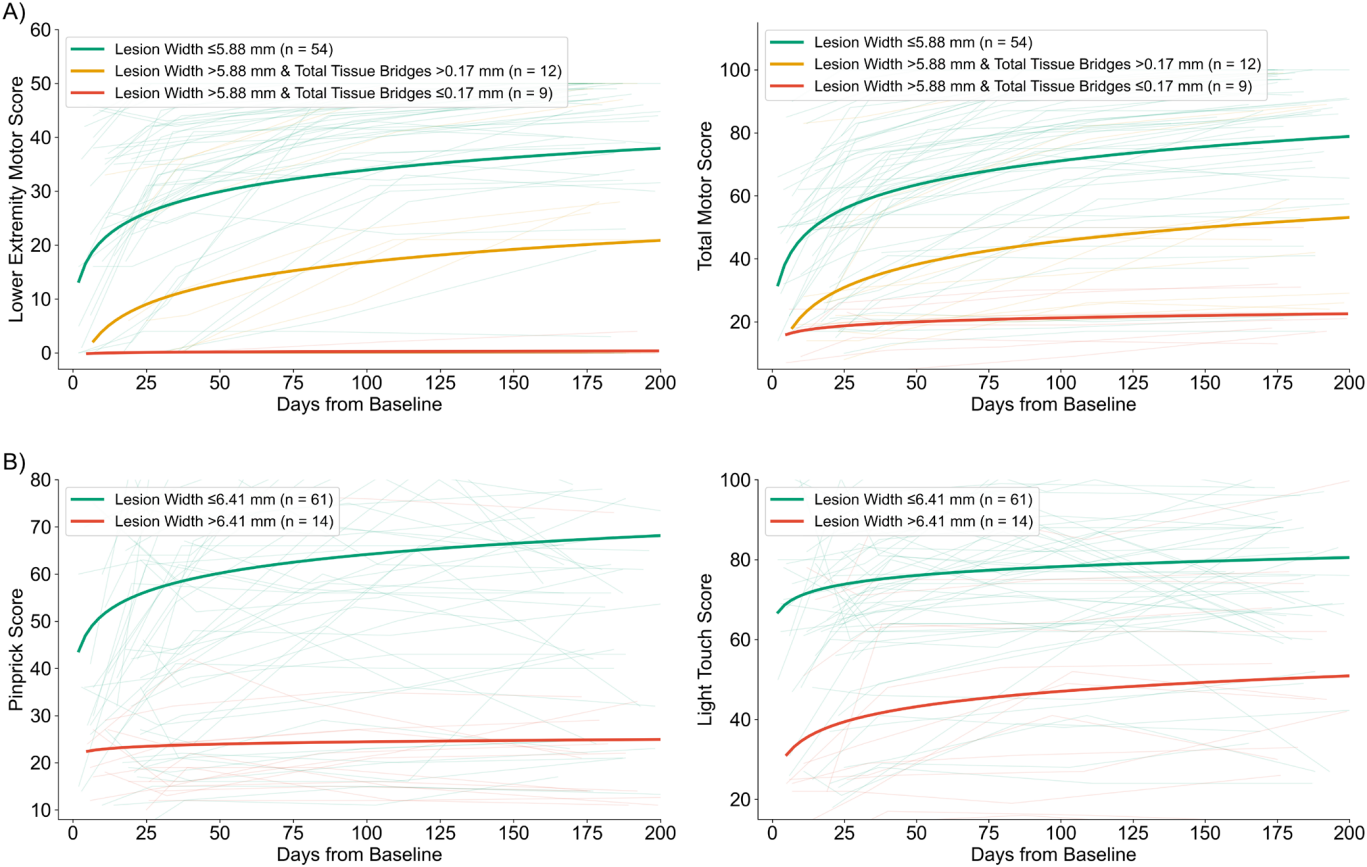
Longitudinal motor and sensory recovery stratified by baseline imaging‐derived lesion measures. Distinct subgroups of (A) motor and (B) sensory recovery trajectories over 6 months post‐injury, stratified by URP‐CTREE model‐derived cut‐offs in automatically derived lesion measures. (A) Lower extremity motor score (LEMS) and total motor score subgroups were stratified into higher and lower recovery by lesion width (≤ 5.88 mm and > 5.88 mm) and total tissue bridges (> 0.17 mm and ≤ 0.17 mm). (B) Pinprick score and light touch score subgroups were stratified into higher and lower recovery by lesion width (≤ 6.41 mm and > 6.41 mm). Thin semi‐transparent lines represent individual participant trajectories, while thick lines show nonlinear mixed‐effects model fits for each group.

## 4 Discussion

In this multicenter longitudinal study of 83 SCI patients from two cohorts with neuroimaging and acute to chronic clinical follow‐up, automatically derived MRI measures of lesion morphometry showed robust agreement with established manual measures, demonstrating comparable prognostic value for neurological recovery after SCI. Notably, lesion width emerged as a key predictor of 6‐month motor and sensory outcomes. Importantly, prediction‐based stratification models yielded similar cut‐offs and subgroup structures for manual and automatic measures, which determined distinct recovery trajectories over the first 6 months post‐SCI.

In our heterogeneous SCI cohort consisting of both tetraplegic and paraplegic patients, sensorimotor scores improved most from baseline to 1‐month, followed by 1‐ to 3‐month and only minimal changes from 3‐ to 6‐month. Neurological recovery plateauing after 3 months post‐injury is consistent with previous SCI literature [29, 30, 31], highlighting this timepoint as a clinically meaningful window during which patients undergo most of the recovery [6]. Similarly, AIS grades improved mainly within the first 3 months. There were more subjects with AIS grade A at 6‐ than 3‐month. In particular, three individuals classified as AIS A at baseline, 1‐month, and 6‐month had deviating grades at 3‐month. This might showcase the pitfalls of the examiner‐dependent ISNCSCI examination [3, 32, 33] and reinforce the application of objective, quantitative biomarkers of lesion severity and post‐SCI recovery.

Automatic lesion morphometrics obtained from conventional T2w MRI scans in this study showed moderate to strong correlations with manual measures, confirming previous research in a smaller cohort of 21 SCI patients [34]. The discrepancy of systematically larger values from manual assessments likely reflects methodological differences inherent to the two approaches. Manual measurements are based on a single midsagittal slice and rely on visual demarcation of lesion and spinal cord borders. This may overestimate the lesion extent due to PVE, midsagittal slice selection, and liberal lesion annotation. In particular, the PVE can be substantial in clinical sagittal T2w scans with a slice thickness of ≥ 3 mm, commonly acquired in SCI patients. In contrast, the automatic pipeline includes intramedullary lesion and spinal cord segmentation using the SCIsegV2 algorithm [14, 15], which incorporates volumetric information and subsequently interpolates midsagittal slices in a soft lesion mask that accounts for PVE, resulting in more conservative estimates. Disagreement in morphometrics might also originate from under‐ or over‐segmentation of SCIseg in some subjects, with the bias accumulating for tissue bridges that depend on the quality of both lesion and spinal cord segmentations. Importantly, despite these absolute differences, automatic measures maintained a similar relative ordering across patients to manual ones and reproduced clinically meaningful cut‐offs, patient stratification, and recovery trajectories. This suggests that a systematic shift is less critical and does not affect the value of lesion morphometrics for outcome prognosis and clinical trial planning, which is essential for translational adoption.

Prediction‐based stratification trees for both manual and automatic measures resulted in similar predictors and cut‐offs, further validating the comparability of the two methods. The consistent identification of lesion width as the primary predictor of motor and sensory recovery across both manual and automatic measurements highlights the importance of transverse injury severity in determining neurological outcome after SCI. This is supported by previous research showing that lesion width is the most powerful metric distinguishing AIS grade converters from non‐converters over 1‐year post‐SCI [23]. A wider lesion likely reflects more extensive bilateral disruption of ascending and descending pathways, limiting the capacity for functional reorganization and recovery [35, 36, 37]. Lesion width cut‐offs of 5.46 mm–7.20 mm for motor and sensory recovery, spanning most of the spinal cord's anteroposterior diameter, were associated with the lowest sensorimotor outcomes at 6 months. Still, a few of the subjects showed higher neurological scores than what their lesion size would suggest. This can most likely be attributed to spared tissue more laterally located [38] and/or intact myotomes/dermatomes with normal motor/sensory function above the NLI [2]. Importantly, we previously showed that NLI does not impact lesion or tissue bridge width, highlighting their robustness as prognostic biomarkers despite NLI‐specific spinal anatomy and biomechanics [23]. To still account for differences in lesion level across subjects, which might impact total motor and sensory scores, we ran URP‐CTREE models with the NLI as an additional variable. While motor outcome trees were confirmed and lesion morphometrics remained the only variables splitting patients, the algorithm identified the NLI in addition to the lesion width for sensory scores, generally distinguishing cervical from thoracolumbar injuries and further improving subgroup separation. Crucially, adding the NLI resolved the previous discrepancy in the lesion width threshold for 6‐month pinprick outcomes between manual and automatic approaches.

In patients with more severe 6‐month motor impairment, additional stratification by automatically derived tissue bridge width further refined outcome prediction, with as little as > 0.17 mm in tissue bridges resulting in significant motor recovery. This underscores the neurobiological relevance of injury‐spared tissue as correlates of residual sensorimotor signal conduction, and aligns with prior work demonstrating that even small amounts of spared tissue can support meaningful neurological improvement and represent a potential window sensitive to interventional treatments [6]. Supported by a previous study revealing a significant role of lesion width and tissue bridge width, but not lesion length, for refined URP‐CTREE stratification of patients into 1‐year AIS grade subgroups [23], this highlights that the post‐SCI recovery potential is primarily determined by both the transverse lesion extent and the preservation of neural tissue encompassing motor and sensory pathways within the injured cord [39, 40]. Tissue bridge width was not found as an additional URP‐CTREE cut‐off for the manual approach, possibly due to the inability to visually detect very small tissue bridges, inaccurate midsagittal slice selection, and subjective overestimation of the lesion. Crucially, the same predictors, corresponding cut‐offs, and subgroup sizes were identified for LEMS and total motor score (for automatic and manual measures), and for pinprick and light touch score (for automatic measures), further demonstrating the consistent prognostic power and clinical relevance of MRI‐derived lesion width and tissue bridges as biomarkers of long‐term outcomes after SCI. Together, these results highlight the robustness and generalizability of automatic lesion morphometrics, further strengthened by our findings of highly comparable stratification cut‐offs observed between a sub‐cohort of patients with imaging > 3 weeks post‐SCI and the full cohort, across motor and sensory outcomes.

In a subsequent data‐driven step, we used the URP‐CTREE‐identified cut‐offs to characterize subgroup‐specific recovery trajectories. The observed longitudinal patterns complement previous AIS grade‐based trajectories [27] and suggest that lesion width is a strong structural determinant of both baseline neurological impairment and subsequent recovery [23], with a similar threshold across motor (5.88 mm) and sensory (6.41 mm) outcomes. Importantly, the additional stratification of motor recovery trajectories by tissue bridge width (0.17 mm) among patients with larger lesions highlights that lesion width alone does not fully capture the recovery potential. Crucially, patients with wide lesions but some spared tissue showed comparable motor recovery rates over 6 months follow‐up to those with a smaller lesion width, despite the offset (i.e., lower scores) at baseline. Conversely, minimal or absent tissue bridges were associated with persistently poor outcomes, reinforcing their relevance as a prognostic marker.

This study has limitations. This was a retrospective study combining data from two SCI cohorts across eight sites acquired over an extended time period. However, despite considerable advances in clinical management and rehabilitation, the neurological and functional recovery after SCI has remained stable over the last two decades [41]. Future research could consider the enrollment date and systematic information on the structure of clinical care and rehabilitation intensity in the recursive partitioning. While we cannot exclude some variability in scanner hardware and acquisition parameters, this heterogeneity enhances generalizability and reflects real‐world clinical conditions under which automatic tools are intended to operate. We have previously demonstrated reliable manual lesion segmentation and tissue bridges quantification across field strengths and MRI vendors [6, 7, 9, 12, 22, 42], and developed the SCIseg algorithm using images acquired at 1.5 T and 3.0 T [14, 15]. Although the identified tissue bridge threshold of 0.17 mm is smaller than the sagittal voxel size, it likely reflects sub‐voxel interpolation between adjacent sagittal slices, which may improve tissue bridge estimation compared with manual measurements. Notably, this threshold was identified in AIS A patients and is consistent with previous reports of manually measured tissue bridges of 0.3 ± 0.7 mm in a multicenter cohort [6]. There were differences in MRI and clinical assessment timepoints across the two cohorts, given the differences in study designs, that could possibly bias the comparison of manual and automatic measurements. However, we showed that T2w‐derived lesions do not undergo significant changes after 2–3 weeks post‐SCI [7], enabling reliable quantification of MRI‐derived measures, and used linear‐mixed effect models for the recovery trajectories to account for the temporal variability. The automatic segmentation method SCIseg was developed on MRI scans spanning different SCI phases [14, 15], thereby providing robust segmentation performance on our cohort. Although a subset of our data were used to develop SCIseg [14, 15], potentially introducing optimism bias, another study conducted on an external cohort showed that SCIseg‐derived morphometrics are consistent with manually assessed measurements [34]. Nonetheless, there remains a risk of circularity for URP‐CTREE‐derived thresholds, which need to be validated in future studies. Although prediction‐based stratification identified robust MRI‐based cut‐offs, the modest sample size of 83 patients with SCI might have limited further subdivision of the resulting subgroups according to demographic variables and independent validation. Prospective studies with larger, harmonized datasets will be necessary to confirm the generalizability and reproducibility of the identified thresholds in our specific cohort as prerequisites for clinical adoption, especially given that some of the subgroups are particularly small. Finally, while this study focused on midsagittal slice‐derived lesion morphometrics, future work should harness the entire output from SCIseg, including 3D metrics (i.e., lesion volume), parasagittal tissue bridges, and axial T2w‐derived measures (e.g., overlap of lesion with distinct spinal tracts using template‐based analysis) [16, 17] and assess their clinical relevance. Moreover, lesion information from conventional T2w MRI might be integrated with quantitative microstructural imaging metrics for remote neuropathological insight and enhanced prognostic accuracy [43, 44].

In summary, automatic MRI‐based quantification of lesion morphometry using SCIseg demonstrates good agreement with manual measurements and, critically, preserves their prognostic relevance for neurological recovery after spinal cord injury. Automatic lesion width and tissue bridges identified comparable cut‐offs to manual assessments, stratified patients into clinically meaningful subgroups of specific outcomes, and delineated distinct recovery trajectories over the first 6 months post‐injury. These findings support automatic lesion morphometry as a scalable and objective imaging biomarker that can replace manual measurements by reducing intra‐ and inter‐rater variability and time burden. This has the potential to facilitate outcome prediction, patient counseling, and multicenter trial design in SCI, advancing the translation of quantitative spinal cord MRI into clinical and research practice.

## Author Contributions

Conception and design of the study: D.P., J.V., J.C.‐A., P.F.; Acquisition and analysis of data: D.P., J.V., L.F., E.N.K., G.D., N.W., R.A., D.M., A.C., M.H.‐G., M.A., M.B.; drafting a significant portion of the manuscript or figures: D.P., J.V., J.C.‐A., P.F. See Data S1 for a list of the Nogo Inhibition in Spinal Cord Injury Study Group and site study personnel and their affiliations.

## Funding

This work was supported by Canada First Research Excellence Fund. Courtois NeuroMod project. Fonds de Recherche du Québec – Santé, 322736, 324636. Natural Sciences and Engineering Research Council of Canada, RGPIN‐2019‐07244. Schweizerischer Nationalfonds zur Förderung der Wissenschaftlichen Forschung, P500PM_214211. H2020 Marie Skłodowska‐Curie Actions, 101107932. Canadian Institutes of Health Research, PJT‐190258, PJT‐203803. Canada Foundation for Innovation, 32454, 34824. Ministerstvo Zdravotnictví České Republiky, NW26J‐04‐00101. Canada Research Chair in Quantitative Magnetic Resonance Imaging, CRC‐2020‐00179.

## Conflicts of Interest

The authors declare no conflicts of interest.

## Supporting information


**Data S1:** The Nogo Inhibition in Spinal Cord Injury Study Group and site study personnel.

**Data S2:** Midsagittal slice interpolation. (A) Algorithm pseudo code. (B) Illustrative example. First, the *lesion's* center of mass along the SI axis is identified from the binary lesion mask (Step 1), and five axial slices around this center of mass (slice at the center of mass and two slices above and two below) are selected (Step 2). For each of these axial slices, the *spinal cord's* center of mass along the LR axis is found from the spinal cord binary mask (Step 3). These coordinates are then averaged to obtain the target position for interpolation in the LR direction (Step 4). Finally, two sagittal slices closest to this position of the lesion and spinal cord masks are interpolated to obtain soft (i.e., non‐binary) masks (Step 5). These soft masks account for the partial volume effect and are used to weight the lesion morphometry measurements.

**Data S3:** Prediction‐based stratification of patients using baseline lesion measures obtained manually and automatically, and the neurological level of injury (NLI). Unbiased recursive partitioning conditional inference tree models for 6‐month (A) motor (i.e., lower extremity and total motor score) and (B) sensory (i.e., pinprick and light touch score) outcomes. Left panel: Trees including manual lesion measures as predictors of sensorimotor outcomes. Right panel: Trees including automatic lesion measures as predictors of sensorimotor outcomes. Consistent measures with similar cut‐offs were identified for both methods, including imaging‐derived lesion morphometrics for 6‐month motor scores and lesion morphometrics and the NLI for 6‐month sensory outcomes. Distribution of 6‐month sensorimotor outcomes is represented by box plots with the median and interquartile range (1st–3rd quartile), and two‐sided error bars. AIS, American Spinal Injury Association Impairment Scale; uk, unknown.

## Data Availability

The developed automatic method is open‐source and freely available through the *sct_analyze_lesion* function as part of the Spinal Cord Toolbox (SCT) v7.0: https://github.com/spinalcordtoolbox/spinalcordtoolbox/releases/tag/7.0. Codes and processing scripts are available at: https://github.com/ivadomed/model_seg_sci/tree/r20260224. The data that support the findings of this study are available from the corresponding author upon reasonable request.

## Appendix

Rainer Abel; Kerstin Rehahn; Mirko Aach; Klaus Röhl; Norbert Weidner; Marion Saur; Nikolaus Weiskopf; Doris Maier; Andreas Badke; Margret Hund‐Georgiadis; Michael Baumberger; Armin Curt; Jesus Benito‐Penalva; Jiri Kriz.

## References

[1] C. S. Ahuja , J. R. Wilson , S. Nori , et al., “Traumatic Spinal Cord Injury,” Nature Reviews Disease Primers 3, no. 1 (2017): 17018, 10.1038/nrdp.2017.18. 28447605

[2] S. C. Kirshblum , S. P. Burns , F. Biering‐Sorensen , et al., “International Standards for Neurological Classification of Spinal Cord Injury (Revised 2011),” Journal of Spinal Cord Medicine 34, no. 6 (2011): 535–546, 10.1179/204577211X13207446293695. PMC3232636 22330108

[3] İ. Gündoğdu , M. Akyüz , E. A. Öztürk , and F. A. Cakcı , “Can Spinal Cord Injury Patients Show a Worsening in ASIA Impairment Scale Classification Despite Actually Having Neurological Improvement? The Limitation of ASIA Impairment Scale Classification,” Spinal Cord 52, no. 9 (2014): 667–670, 10.1038/sc.2014.89. 24891005

[4] G. David , S. Mohammadi , A. R. Martin , et al., “Traumatic and Nontraumatic Spinal Cord Injury: Pathological Insights From Neuroimaging,” Nature Reviews. Neurology 15, no. 12 (2019): 718–731, 10.1038/s41582-019-0270-5. 31673093

[5] P. Freund , M. Seif , N. Weiskopf , et al., “MRI in Traumatic Spinal Cord Injury: From Clinical Assessment to Neuroimaging Biomarkers,” Lancet Neurology 18, no. 12 (2019): 1123–1135, 10.1016/S1474-4422(19)30138-3. 31405713

[6] D. Pfyffer , A. C. Smith , K. A. Weber, 2nd , et al., “Prognostic Value of Tissue Bridges in Cervical Spinal Cord Injury: A Longitudinal, Multicentre, Retrospective Cohort Study,” Lancet Neurology 23, no. 8 (2024): 816–825, 10.1016/s1474-4422(24)00173-x. PMC12128092 38945142

[7] D. Pfyffer , E. Huber , R. Sutter , A. Curt , and P. Freund , “Tissue Bridges Predict Recovery After Traumatic and Ischemic Thoracic Spinal Cord Injury,” Neurology 93, no. 16 (2019): e1550–e1560, 10.1212/WNL.0000000000008318. PMC6815206 31541012

[8] B. Aarabi , C. A. Sansur , D. M. Ibrahimi , et al., “Intramedullary Lesion Length on Postoperative Magnetic Resonance Imaging Is a Strong Predictor of ASIA Impairment Scale Grade Conversion Following Decompressive Surgery in Cervical Spinal Cord Injury,” Neurosurgery 80, no. 4 (2017): 610–620, 10.1093/neuros/nyw053. PMC5748932 28362913

[9] E. Huber , P. Lachappelle , R. Sutter , A. Curt , and P. Freund , “Are Mid‐ Sagittal Tissue Bridges Predictive of Outcome After Cervical Spinal Cord Injury?,” Annals of Neurology 81 (2017): 740–748, 10.1002/ana.24932. 28393423

[10] D. R. O'Dell , K. A. Weber , J. C. Berliner , et al., “Midsagittal Tissue Bridges Are Associated With Walking Ability in Incomplete Spinal Cord Injury: A Magnetic Resonance Imaging Case Series,” Journal of Spinal Cord Medicine 43, no. 2 (2020): 268–271. 10.1080/10790268.2018.1527079 PMC7054908 30346248

[11] W. A. Thornton , K. Smulligan , K. A. Weber, 2nd , et al., “Lesion Characteristics Are Associated With Bowel, Bladder, and Overall Independence Following Cervical Spinal Cord Injury,” Journal of Spinal Cord Medicine 48, no. 5 (2025): 858–866, 10.1080/10790268.2024.2363005. PMC12329833 38958637

[12] D. Pfyffer , K. Vallotton , A. Curt , and P. Freund , “Predictive Value of Midsagittal Tissue Bridges on Functional Recovery After Spinal Cord Injury,” Neurorehabilitation and Neural Repair 35, no. 1 (2021): 33–43, 10.1177/1545968320971787. PMC8350965 33190619

[13] N. Weidner , R. Abel , D. Maier , et al., “Safety and Efficacy of Intrathecal Antibodies to Nogo‐A in Patients With Acute Cervical Spinal Cord Injury: A Randomised, Double‐Blind, Multicentre, Placebo‐Controlled, Phase 2b Trial,” Lancet Neurology 24, no. 1 (2025): 42–53, 10.1016/S1474-4422(24)00447-2. 39706632

[14] E. N. Karthik , J. Valošek , A. C. Smith , et al., “SCIseg: Automatic Segmentation of Intramedullary Lesions in Spinal Cord Injury on T2‐Weighted MRI Scans,” Radiology: Artificial Intelligence 7, no. 1 (2025): e240005, 10.1148/ryai.240005. PMC11791505 39503603

[15] E. N. Karthik , J. Valošek , L. Farner , et al., “SCIsegV2: A Universal Tool for Segmentation of Intramedullary Lesions in Spinal Cord Injury,” in Lecture Notes in Computer Science (Springer Nature Switzerland, 2025), 198–209.

[16] S. Schading‐Sassenhausen , V. Dietz , and P. Freund , “Effect of Corticospinal and Reticulospinal Tract Damage on Spastic Muscle Tone and Mobility: A Retrospective Observational MRI Study,” eBioMedicine 118 (2025): 105824, 10.1016/j.ebiom.2025.105824. PMC12256215 40561563

[17] L. Farner , T. M. Emmenegger , S. Schading‐Sassenhausen , et al., “3D MRI Tract‐Specific Spinal Cord Lesion Pattern Improves Prediction of Distinct Neurological Recovery,” Annals of Clinical and Translational Neurology 12 (2025): 1762–1770, 10.1002/acn3.70087. PMC12455861 40574619

[18] B. De Leener , S. Lévy , S. M. Dupont , et al., “SCT: Spinal Cord Toolbox, an Open‐Source Software for Processing Spinal Cord MRI Data,” NeuroImage 145 (2017): 24–43, 10.1016/j.neuroimage.2016.10.009. 27720818

[19] K. J. Gorgolewski , T. Auer , V. D. Calhoun , et al., “The Brain Imaging Data Structure, a Format for Organizing and Describing Outputs of Neuroimaging Experiments,” Scientific Data 3, no. 1 (2016): 160044, 10.1038/sdata.2016.44. PMC4978148 27326542

[20] P. Virtanen , R. Gommers , and T. E. Oliphant , “SciPy 1.0: Fundamental Algo‐ Rithms for Scientific Computing in Python,” Nature Methods 17, no. 3 (2020): 261–272. 10.1038/s41592-019-0686-2 PMC7056644 32015543

[21] T. Hothorn , K. Hornik , and A. Zeileis , “Unbiased Recursive Partitioning: A Conditional Inference Framework,” Journal of Computational and Graphical Statistics 15, no. 3 (2006): 651–674, 10.1198/106186006X133933.

[22] D. Pfyffer , K. Vallotton , A. Curt , and P. Freund , “Tissue Bridges Predict Neuropathic Pain Emergence After Spinal Cord Injury,” Journal of Neurology, Neurosurgery, and Psychiatry 91, no. 10 (2020): 1111–1117, 10.1136/jnnp-2020-323150. PMC7509517 32788257

[23] S. Schading‐Sassenhausen , D. Pfyffer , L. Farner , et al., “Extent of Traumatic Spinal Cord Injury Is Lesion Level Dependent and Predictive of Recovery: A Multicenter Neuroimaging Study,” Journal of Neurotrauma 41, no. 17–18 (2024): 2146–2157, 10.1089/neu.2023.0555. PMC12054703 39001825

[24] L. G. Tanadini , T. Hothorn , L. A. T. Jones , et al., “Toward Inclusive Trial Protocols in Heterogeneous Neurological Disorders: Prediction‐Based Stratification of Participants With Incomplete Cervical Spinal Cord Injury,” Neurorehabilitation and Neural Repair 29, no. 9 (2015): 867–877. 10.1177/1545968315570322 25644238

[25] N. Evaniew , N. Fallah , C. S. Rivers , et al., “Unbiased Recursive Partitioning to Stratify Patients With Acute Traumatic Spinal Cord Injuries: External Validity in an Observational Cohort Study,” Journal of Neurotrauma 36, no. 18 (2019): 2732–2742, 10.1089/neu.2018.6335. PMC6727480 30864876

[26] I.‐M. Velstra , M. Bolliger , L. G. Tanadini , et al., “Prediction and Stratification of Upper Limb Function and Self‐Care in Acute Cervical Spinal Cord Injury With the Graded Redefined Assessment of Strength, Sensibility, and Prehension (GRASSP),” Neurorehabilitation and Neural Repair 28, no. 7 (2014): 632–642, 10.1177/1545968314521695. 24566986

[27] A. Curt , H. J. A. Van Hedel , D. Klaus , et al., “Recovery From a Spinal Cord Injury: Significance of Compensation, Neural Plasticity, and Repair,” Journal of Neurotrauma 25, no. 6 (2008): 677–685, 10.1089/neu.2007.0468. 18578636

[28] S. Schading , G. David , T. M. Emmenegger , et al., “Dynamics of Progressive Degeneration of Major Spinal Pathways Following Spinal Cord Injury: A Longitudinal Study,” NeuroImage: Clinical 37 (2023): 103339, 10.1016/j.nicl.2023.103339. PMC9939725 36758456

[29] J. D. Steeves , D. Lammertse , A. Curt , et al., “Guidelines for the Conduct of Clinical Trials for Spinal Cord Injury (SCI) as Developed by the ICCP Panel: Clinical Trial Outcome Measures,” Spinal Cord 45, no. 3 (2007): 206–221, 10.1038/sj.sc.3102008. 17179972

[30] C. H. Merritt , M. A. Taylor , C. J. Yelton , and S. K. Ray , “Economic Impact of Traumatic Spinal Cord Injuries in the United States,” Neuroimmunology and Neuroinflammation 6 (2019): 9, 10.20517/2347-8659.2019.15. PMC8052100 33869674

[31] M. Henry , R. C. Coxe , A. Barry , et al., “A Research Protocol to Study the Critical Time Window for Rehabilitation After Incomplete Spinal Cord Injury: Early vs. Late Locomotor Training,” BMC Neurology 24, no. 1 (2024): 482, 10.1186/s12883-024-03980-x. PMC11657539 39702102

[32] H. A. Hong , J. Parsons , J. Xu , et al., “An Evaluation of International Standards for Neurological Classification of Spinal Cord Injury (ISNCSCI) Performance Within the Canadian SCI Network,” Topics in Spinal Cord Injury Rehabilitation 31, no. 3 (2025): 15–36, 10.46292/sci25-00011. PMC12376162 40873963

[33] C. Schuld , S. Franz , L. Heutehaus , et al., “Clarifications of the Motor Level Definition in the International Standards for Neurological Classification of Spinal Cord Injury in Not Clinically Testable Myotomes,” Topics in Spinal Cord Injury Rehabilitation 31, no. 3 (2025): 37–47, 10.46292/sci24-00094. PMC12376153 40873965

[34] J. R. Connor , W. A. Thornton , K. A. Weber , et al., “Reliability of SCIseg Automated Measurement of Midsagittal Tissue Bridges in Spinal Cord Injuries Using an External Dataset,” Topics in Spinal Cord Injury Rehabilitation 31, no. 2 (2025): 39–49, 10.46292/sci25-00015. PMC12199566 40585009

[35] P. Freund , E. Schmidlin , T. Wannier , et al., “Nogo‐A‐Specific Antibody Treatment Enhances Sprouting and Functional Recovery After Cervical Lesion in Adult Primates,” Nature Medicine 12, no. 7 (2006): 790–792, 10.1038/nm1436. 16819551

[36] O. Raineteau and M. E. Schwab , “Plasticity of Motor Systems After Incomplete Spinal Cord Injury,” Nature Reviews. Neuroscience 2, no. 4 (2001): 263–273, 10.1038/35067570. 11283749

[37] F. M. Bareyre , M. Kerschensteiner , O. Raineteau , T. C. Mettenleiter , O. Weinmann , and M. E. Schwab , “The Injured Spinal Cord Spontaneously Forms a New Intraspinal Circuit in Adult Rats,” Nature Neuroscience 7, no. 3 (2004): 269–277, 10.1038/nn1195. 14966523

[38] X. Hu , W. Xu , Y. Ren , et al., “Spinal Cord Injury: Molecular Mechanisms and Therapeutic Interventions,” Signal Transduction and Targeted Therapy 8, no. 1 (2023): 245, 10.1038/s41392-023-01477-6. PMC10291001 37357239

[39] A. Singh , S. Balasubramanian , M. Murray , M. Lemay , and J. Houle , “Role of Spared Pathways in Locomotor Recovery After Body‐Weight‐Supported Treadmill Training in Contused Rats,” Journal of Neurotrauma 28, no. 12 (2011): 2405–2416, 10.1089/neu.2010.1660. PMC3235344 21568686

[40] N. Punjani , D. Deska‐Gauthier , L. D. Hachem , et al., “Neuroplasticity and Regeneration After Spinal Cord Injury,” North American Spine Society Journal 15 (2023): 100235. 10.1016/j.xnsj.2023.100235 PMC10320621 37416090

[41] L. Bourguignon , B. Tong , F. Geisler , et al., “International Surveillance Study in Acute Spinal Cord Injury Confirms Viability of Multinational Clinical Trials,” BMC Medicine 20, no. 1 (2022): 225, 10.1186/s12916-022-02395-0. PMC9202190 35705947

[42] K. Vallotton , E. Huber , R. Sutter , A. Curt , M. Hupp , and P. Freund , “Width and Neurophysiologic Properties of Tissue Bridges Predict Recovery After Cervical Injury,” Neurology 92, no. 24 (2019): e2793–e2802, 10.1212/WNL.0000000000007642. PMC6598793 31092621

[43] G. David , M. Seif , E. Huber , et al., “In Vivo Evidence of Remote Neural Degeneration in the Lumbar Enlargement After Cervical Injury,” Neurology 92, no. 12 (2019): e1367–e1377, 10.1212/WNL.0000000000007137. PMC6511094 30770423

[44] G. David , D. Pfyffer , K. Vallotton , et al., “Longitudinal Changes of Spinal Cord Grey and White Matter Following Spinal Cord Injury,” Journal of Neurology, Neurosurgery, and Psychiatry 92, no. 11 (2021): 1222–1230, 10.1136/jnnp-2021-326337. PMC8522459 34341143

