# Supplementary material for: Automatic Lesion Morphometrics Reliably Predict Sensorimotor Recovery After Spinal Cord Injury: A Longitudinal, Multicenter Neuroimaging Study
Source: Ann Clin Transl Neurol. 2026 Nov 5:10.1002/acn3.70569. Online ahead of print. doi: 10.1002/acn3.70569 (PMC13739854; doi:10.1002/acn3.70569)
Supplement: Supplementary file 1 [file ACN3-9999-0-s001.docx]

**Algorithm 1: Midsagittal slice interpolation.**

(A) Algorithm pseudo code. (B) Illustrative example. First, the *lesion’s* center of mass along the SI axis is identified from the binary lesion mask (Step 1), and five axial slices around this center of mass (slice at the center of mass and two slices above and two below) are selected (Step 2). For each of these axial slices, the *spinal cord’s* center of mass along the LR axis is found from the spinal cord binary mask (Step 3). These coordinates are then averaged to obtain the target position for interpolation in the LR direction (Step 4). Finally, two sagittal slices closest to this position of the lesion and spinal cord masks are interpolated to obtain soft (i.e., non-binary) masks (Step 5). These soft masks account for the partial volume effect and are used to weight the lesion morphometry measurements.
