# Supplementary material for: Automatic Lesion Morphometrics Reliably Predict Sensorimotor Recovery After Spinal Cord Injury: A Longitudinal, Multicenter Neuroimaging Study
Source: Ann Clin Transl Neurol. 2026 Nov 5:10.1002/acn3.70569. Online ahead of print. doi: 10.1002/acn3.70569 (PMC13739854; doi:10.1002/acn3.70569)
Supplement: Supplementary file 2 [file ACN3-9999-0-s002.docx]

**The Nogo Inhibition in Spinal Cord Injury Study Group and site study personnel:**
Principal Site Investigators are underlined.

**Germany**

*Bayreuth*: Rainer Abel M.D., Katja-Kerstin Bössl M.D., Anika Steger

*Berlin*: Sandra Arndt, Malte Gössling M.D., Martin Kreuzträger M.D., Thomas Liebscher M.D., Franziska Radach, Kerstin Rehahn M.D.

*Bochum*: Mirko Aach M.D., Katalin Barkovits, Ph.D., Dennis Grasmücke M.D., Manuela Klettke, Katrin Marcus Ph.D.

*Halle*: Gudrun Allmendinger, Carolin Gräbsch, Frank Röhrich M.D., Klaus Röhl M.D., F. W. Weidt M.D.

*Heidelberg*: Ina Burghaus Ph.D., Stefan Fichtner, Steffen Franz M.D., Cornelia Hensel M.D., Laura Heutehaus M.Sc., Johannes Hüsing Ph.D., Andreas Hug M.D., Naemi Kühn Ph.D., Annette Langpape, AntoniaSophie Luz, Melanie Motsch, Radhika Puttagunta Ph.D., Christoph Rehnitz M.D., Rüdiger Rupp Ph.D., Christian Schuld M.Sc., Dominik Teichert Ph.D., Björn Wagner M.D., Norbert Weidner M.D., Tanja Weis Ph.D.

*Hessisch-Lichtenau*: Nadine Rohleder, Marion Saur M.D., Josina Waldmann M.D.

*Leipzig*: Tobias Leutritz Ph.D., Nikolaus Weiskopf Ph.D.

*Murnau*: Lukas Grassner M.D., Orpheus Mach, Doris Maier M.D., Ludwig Sanktjohanser M.D., Matthias Vogel M.D.

*Tübingen*: Andreas Badke M.D., Elke Kirsch, Marie Reumann M.D.

**Switzerland**

*Basel*: Vera Bouverat M.D., Isabelle Debecker, Kerstin Hug M.D., Margret Hund-Georgiadis M.D., Holger Lochmann M.D., Elena Pauli

*Bern*: Petra Zalud, Ph.D.

*Nottwil*: Michael Baumberger M.D., Desiree Beck, Agata Bulloni, Angela Frotzler Ph.D., Evelyn Rickenbacher, Anke Scheel M.D., Jürgen Schneider M.D.

*Zürich*: Marc Bolliger Ph.D., Armin Curt M.D., Lynn Farner M.Sc., Patrick Freund M.D., Torsten Hothorn Ph.D., Michèle Hubli Ph.D., Tim Killeen M.D., Iris Krüsi, Michael A. Maurer Ph.D., Andrea Prusse, Paulina S. Scheuren Ph.D., Martin Schubert M.D., Martin E. Schwab Ph.D., Maryam Seif, Christina Sina Ph.D., Bettina Steiner M.Sc.

**Spain**

*Barcelona*: Jesus Benito-Penalva M.D., Hatice Kumru M.D., Josep Medina Ph.D., Eloy Opisso Ph.D., Joan Vidal M.D.

**Czechia**

*Prague*: Renata Hakova M.D., Veronika Hysperska M.D., Jiri Kriz M.D.
